# Prevalence of iron deficiency in 62,685 women of seven race/ethnicity groups: The HEIRS Study

**DOI:** 10.1371/journal.pone.0232125

**Published:** 2020-04-23

**Authors:** James C. Barton, Howard H. Wiener, Ronald T. Acton, Paul C. Adams, John H. Eckfeldt, Victor R. Gordeuk, Emily L. Harris, Christine E. McLaren, Helen Harrison, Gordon D. McLaren, David M. Reboussin

**Affiliations:** 1 Department of Medicine, University of Alabama at Birmingham, Birmingham, AL, USA and Southern Iron Disorders Center, Birmingham, AL, United States of America; 2 Department of Epidemiology, School of Public Health, University of Alabama at Birmingham, Birmingham, AL, United States of America; 3 USA and Southern Iron Disorders Center, Department of Microbiology, University of Alabama at Birmingham, Birmingham, AL, United States of America; 4 Department of Medicine, London Health Sciences Centre, London, ONT, Canada; 5 Department of Laboratory Medicine and Pathology, University of Minnesota, Minneapolis, MN, United States of America; 6 Division of Hematology and Oncology, Department of Medicine, University of Illinois at Chicago, Chicago, IL, United States of America; 7 Division of Cancer Control and Population Sciences, Epidemiology and Genomics Research Program, National Cancer Institute, National Institutes of Health, Bethesda, MD, United States of America; 8 Department of Epidemiology, University of California, Irvine, CA, United States of America; 9 The Western-Fanshawe Collaborative BScN Program, Fanshawe College, London, ONT, Canada; 10 Division of Hematology/Oncology, Department of Medicine, University of California, Irvine, CA, USA and Department of Veterans Affairs Long Beach Healthcare System, Long Beach, CA, United States of America; 11 Department of Biostatistical Sciences, Wake Forest School of Medicine, Winston-Salem, NC, United States of America; Pennsylvania State University College of Medicine, UNITED STATES

## Abstract

**Background:**

Few cross-sectional studies report iron deficiency (ID) prevalence in women of different race/ethnicity and ages in US or Canada.

**Materials and methods:**

We evaluated screening observations on women who participated between 2001–2003 in a cross-sectional, primary care-based sample of adults ages ≥25 y whose observations were complete: race/ethnicity; age; transferrin saturation; serum ferritin; and *HFE* p.C282Y and p.H63D alleles. We defined ID using a stringent criterion: combined transferrin saturation <10% and serum ferritin <33.7 pmol/L (<15 μg/L). We compared ID prevalence in women of different race/ethnicity subgrouped by age and determined associations of p.C282Y and p.H63D to ID overall, and to ID in women ages 25–44 y with or without self-reported pregnancy.

**Results:**

These 62,685 women included 27,079 whites, 17,272 blacks, 8,566 Hispanics, 7,615 Asians, 449 Pacific Islanders, 441 Native Americans, and 1,263 participants of other race/ethnicity. Proportions of women with ID were higher in Hispanics and blacks than whites and Asians. Prevalence of ID was significantly greater in women ages 25–54 y of all race/ethnicity groups than women ages ≥55 y of corresponding race/ethnicity. In women ages ≥55 y, ID prevalence did not differ significantly across race/ethnicity. p.C282Y and p.H63D prevalence did not differ significantly in women with or without ID, regardless of race/ethnicity, age subgroup, or pregnancy.

**Conclusions:**

ID prevalence was greater in Hispanic and black than white and Asian women ages 25–54 y. p.C282Y and p.H63D prevalence did not differ significantly in women with or without ID, regardless of race/ethnicity, age subgroup, or pregnancy.

## Introduction

Iron deficiency (ID) in women is a cosmopolitan health and nutritional problem [1]. ID in women is associated with fatigue, impaired muscular performance, decreased ability to maintain body temperature on exposure to cold, mucosal and epithelial abnormalities, pica, disturbances in menstruation [2], adverse pregnancy outcomes [3, 4], and decreased mental development of children [5]. The estimated prevalence of ID is lower in women in North America than Africa, Asia, Europe, Latin America and the Caribbean, and Oceania [6], but few cross-sectional studies report the prevalence of ID in women of different race/ethnicity and age who reside in North America [7, 8].

Hemochromatosis is a group of heritable disorders characterized by increased iron absorption, elevated body iron stores, and increased risk for cirrhosis, diabetes, hypogonadism, arthropathy, and bacterial infection [9, 10]. The hemochromatosis gene *HFE* ("high-iron") is linked to the major histocompatibility complex on chromosome 6p [11]. *HFE* encodes the major histocompatibility complex class I-like protein HFE that binds beta-2 microglobulin [12]. HFE protein influences iron absorption by modulating the expression of hepcidin, the main controller of iron metabolism [13]. There are two common missense mutations of *HFE*: p.C282Y (rs1800562) and p.H63D (rs179945) [11, 14]. Whereas p.C282Y occurs predominantly in European whites, p.H63D is cosmopolitan [15]. Among whites of European ancestry who have hemochromatosis phenotypes, approximately 90% are p.C282Y homozygotes [9, 10]. Approximately 10% of European whites are p.C282Y heterozygotes and 24% are p.H63D heterozygotes [9, 10, 16]. It has been hypothesized that hemochromatosis heterozygosity, later defined as p.C282Y heterozygosity, increases iron absorption and thereby decreases risk of ID, especially in women [17, 18].

To learn more, we evaluated initial screening observations on 62,685 women who participated in the Hemochromatosis and Iron Overload Screening Study (HEIRS Study), a cross-sectional, multi-ethnic, primary care-based sample of 101,168 adults ages ≥25 y enrolled during a two-year interval at five Field Centers in the US and Canada [19]. Initial screening observations included age, self-identified race/ethnicity, measurement of transferrin saturation (TS) and serum ferritin (SF), *HFE* p.C282Y and p.H63D genotyping, and self-reported pregnancy. We defined ID as combined initial screening TS <10% and SF <33.7 pmol/L (<15 μg/L), consistent with previous studies [1, 2, 20, 21]. We compared the prevalence of ID in women of different race/ethnicity subgrouped by age. We also determined relationships of ID to p.C282Y and p.H63D, with or without self-reported pregnancy. We discuss our findings in the context of other reports of the prevalence of and risk factors for ID in women who reside in the US or Canada.

## Materials and methods

### Subjects

The National Institutes of Health HEIRS Study (January 2000-January 2006) evaluated the prevalence, genetic and environmental determinants, and potential clinical, personal, and societal effects of hemochromatosis and iron overload in a multi-ethnic, primary care-based sample of 101,168 adults enrolled during a two-year interval (2001–2003) at four Field Centers in the US (Birmingham, Alabama; Irvine, California; Washington, D.C.; and Portland, Oregon/Honolulu, Hawaii) and one in Canada (London, Ontario) [19].

This Study was conducted in accordance with the principles of the Declaration of Helsinki. The local Institutional Review Board of each Field Center approved the Study protocol [5, 9]. The Field Center Institutional Review Boards included: UAB Institutional Review Board for Human Use (Birmingham, Alabama); UC Irvine Institutional Review Board (Irvine, California); Howard University Institutional Review Board (Washington, D.C.); Kaiser Permanente Northwest Region IRB (Portland, Oregon); Kaiser Permanente Hawaii Region IRB (Honolulu, Hawaii); and University of Western Ontario Research Ethics Board for Health Sciences Research Involving Human Subjects (London, Ontario). Participants, ≥25 y of age who were able to give written informed consent, were recruited from a health maintenance organization, diagnostic blood collection centers, and public and private primary care offices in ambulatory clinics associated with the Field Centers. Initial screening included TS and SF phenotyping and *HFE* genotyping (p.C282Y (rs1800562) and p.H63D (rs179945) alleles) [5, 9].

Race/ethnicity was determined by self-reported answers to two questions: one regarding Hispanic background; and another offering a non-exclusive choice of five race/ethnicity groups. Participants who identified themselves as having Hispanic, Latino, or Spanish heritage were classified as Hispanic, regardless of additional race/ethnicity identification or Field Center. Participants who self-identified themselves as non-Hispanic white or Caucasian were classified as whites. Participants classified as blacks included self-identified African Americans/non-Hispanic blacks recruited at US Field Centers (especially central Alabama (University of Alabama at Birmingham) and Washington, D.C. (Howard University)) and self-identified black, African, Haitian, Jamaican, or Somali participants recruited at the Ontario Field Center only. Self-identified Asians were so classified. Participants who reported any Hawaiian or other Pacific Islander heritage were classified as Pacific Islanders. Participants who identified themselves as American Indian or Alaska Native at US Field Centers (or North American Indian, Metis, or Inuit at the Ontario Field Center only) were classified as Native Americans [19, 22]. Participants who reported two or more race/ethnicity groups or unknown race/ethnicity were classified as Other.

Self-reported pregnancy data were obtained from an initial screening question with three possible responses: pregnant; not sure; or not pregnant. For these analyses, we excluded participants who answered "not sure."

We excluded participants who reported learning of the HEIRS Study from a participating family member or who reported on their screening questionnaire that they had been previously diagnosed to have hemochromatosis or iron overload [19]. We also excluded participants with incomplete data pertinent to the present study.

### Laboratory methods

Blood samples were obtained at initial screening for measurement of TS and SF (without regard for state of fasting) and *HFE* mutation analysis [22]. The Central Laboratory (located at Advanced Research and Diagnostic Laboratory at the University of Minnesota, Minneapolis, MN) performed all screening tests, except TS testing of London Health Sciences Centre participants (performed by MDS Laboratory Services, Canada, using an almost identical method). Measurements included spectrophotometric serum iron and unsaturated iron-binding capacity, turbidometric immunoassay of SF (Roche Diagnostics/Hitachi 911, Indianapolis, IN, USA), and calculated total iron-binding capacity and TS [19]. Analytical variability was determined for TS and SF by analysis of routine internal laboratory quality control pools in each analytical batch. For TS measurements, the batch-to-batch coefficient of variation was 3.0%. The correlation coefficient of original participant values and blind replicates was 0.98. For SF measurements, the batch-to-batch coefficient of variation was 4.7%. The correlation coefficient of original participant values and blind replicates was 0.99 [23].

*HFE* p.C282Y and p.H63D were detected as reported in detail elsewhere [19, 23]. Specimens for which initial results were inconclusive were evaluated further using a different method to resolve uncertainty about genotypes [23].

### Definition of iron deficiency

We defined ID as the combination of initial screening TS <10% and initial screening SF <33.7 pmol/L (<15 μg/L), consistent with previous studies [1, 2, 20, 21].

### Statistics

We analyzed observations on 62,685 women who underwent initial HEIRS Study screening for whom these data were complete: race/ethnicity, age, TS, SF, and *HFE* alleles. We also analyzed association of self-reported pregnancy (and self-reported non-pregnancy) and *HFE* alleles in women ages 25–44 y with ID. Most descriptive data are displayed as enumerations, percentages, or means ± 1 standard deviation. Percentages were compared using the likelihood ratio chi-square test or Fisher’s exact test (two-tailed), as appropriate. Mean values were compared using Student t-test (two-tailed) for two groups or F statistic from a linear model for more than two groups. We also compared ages of race/ethnicity groups using an analysis of variance (ANOVA) test. Values of p <0.05 were defined as significant. Analyses were performed with SAS v. 9.0 (SAS Institute, Cary, NC, USA), Excel 2000^®^ (Microsoft Corp., Redmond, WA, USA), and GB-Stat^®^ (v. 10.0, 2003, Dynamic Microsystems, Inc., Silver Spring, MD, USA).

## Results

### Iron deficiency in race/ethnicity groups

There were significant differences between proportions of women with ID across race/ethnicity groups (p <0.0001, likelihood ratio chi-square = 334.84 with 6 degrees of freedom) and mean age by race/ethnicity (p <0.0001, F statistic = 363.37 with 6 numerator degrees of freedom and 62,678 denominator degrees of freedom) (Table 1). A comparison of the prevalence of ID in the first two groups (Hispanic and black) vs. the second two groups (white and Asian) yielded a Fisher's exact test value of p <0.0001, ignoring the three smallest groups (Pacific Islander, Native American, and Other) (Table 1). Comparisons of the mean ages of the same two respective groups yielded p <0.0001 (ANOVA test). Proportions of Hispanic and black women with ID were significantly higher than those of white or Asian women. Proportions of women classified as Pacific Islander, Native American, and Other race/ethnicity with ID were lower than those of white or Asian women, although the overall numbers of women with Pacific Islander, Native American, and Other race/ethnicity were low (Table 1).

**Table 1:** Iron deficiency (ID) in 62,685 women in the HEIRS study^a^.

Race/ethnicity (n)	FeDef, % (n)	Mean age, y (± 1 SD)
Hispanic (8,566)	5.14 (440)	43.91 ± 13.03
Black (17,272)	4.31 (744)	48.69 ± 14.48
White (27,079)	1.99 (539)	51.81 ± 14.22
Asian (7,615)	2.10 (160)	50.04 ± 13.50
Pacific Islander (449)	3.12 (14)	51.82 ± 13.96
Native American (441)	5.22 (23)	47.61 ± 13.97
Other^b^ (1,263)	2.93 (37)	49.84 ± 13.50

^a^ID was defined as combined initial screening transferrin saturation <10% and initial screening serum ferritin <15 μg/L.

^b^Participants who reported two or more race/ethnicity groups or unknown race/ethnicity were classified as Other.

### Iron deficiency by age subgroup

**Fig 1:**
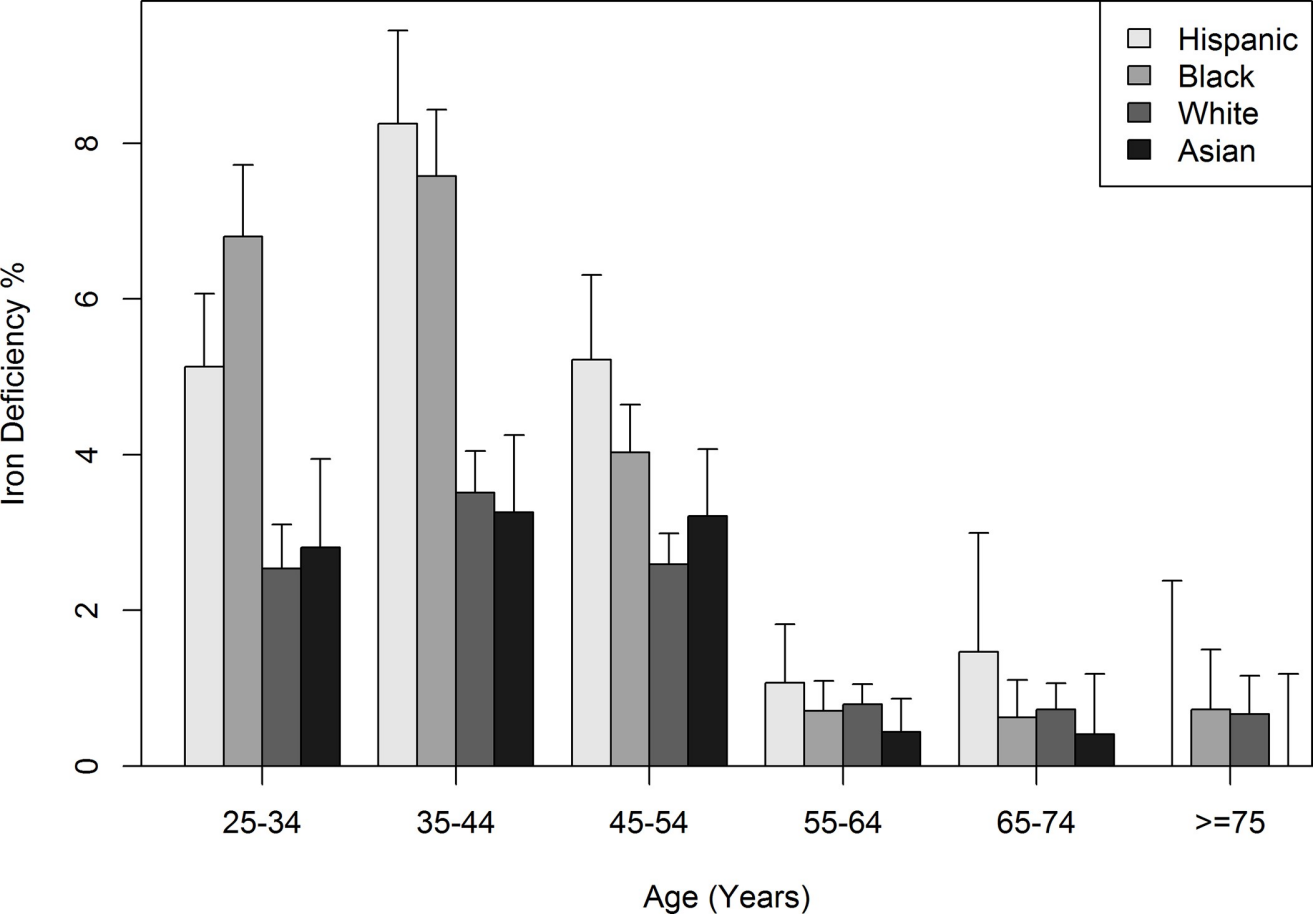
Frequency histogram of prevalence of iron deficiency (mean ± 1 standard deviation) in 62,685 women. These data represent women who participated in the Hemochromatosis and Iron Overload Screening Study.

ID was more prevalent in Hispanic, black, white, and Asian women ages 25–54 y than in women ages ≥55 y of each respective race/ethnicity (Fig 1). The prevalence of ID in Hispanic and black women ages 25–54 y was greater than that of white or Asian women ages 25–54 y (p <0.0001 for both the likelihood ratio test using all race/ethnicity groups, and Fisher's exact test using two race/ethnicity groups indicated above). In women ages ≥55 y, differences in the prevalence of ID across Hispanic, black, white, and Asian groups were not significant (p = 0.095 based on likelihood ratio test using all race/ethnicity groups and p = 0.394 based on Fisher's exact test using combinations of two race/ethnicity groups) (Fig 1).

Of 14 Pacific Islander women with ID, 12 (85.7%) were ages 25–54 y. Of 23 Native American women with ID, 22 (95.7%) were ages 25–54 y. Of 37 women classified as Other race/ethnicity, 36 (97.3%) were ages 25–54 y.

### Iron deficiency and *HFE* p.C282Y and p.H63D

The prevalence of *HFE* p.C282Y and p.H63D alleles did not differ significantly in women with or without ID, regardless of race/ethnicity or age subgroup (Table 2). The prevalence of p.C282Y homozygosity did not differ significantly in women with or without ID, regardless of race/ethnicity or age subgroup. In comparisons of zero alleles vs. one allele similar to those displayed in Table 2, we observed no significant differences (data not shown).

**Table 2:** Prevalence of iron deficiency in women by *HFE* p.C282Y and p.H63D alleles^a^.

Race	Age, y	*HFE* allele	0 alleles, % (n subjects)	Any allele, % (n subjects)	Value of p (0 vs. any alleles)
Hispanic	25–54	p.C282Y	6.19 (6,528)	8.47 (177)	0.2076
Hispanic	25–54	p.H63D	6.19 (5,473)	6.49 (1,232)	0.6960
Hispanic	≥55	p.C282Y	1.13 (1,771)	0.00 (61)	1.0000
Hispanic	≥55	p.H63D	1.22 (1,475)	0.56 (357)	0.3990
Black	25–54	p.C282Y	6.00 (11,486)	7.51 (253)	0.2874
Black	25–54	p.H63D	6.01 (11,091)	6.33 (648)	0.7340
Black	≥55	p.C282Y	0.65 (5,370)	0.70 (142)	0.6104
Black	≥55	p.H63D	0.68 (5,176)	0.30 (336)	0.7233
White	25–54	p.C282Y	2.95 (13,666)	2.69 (1,747)	0.5975
White	25–54	p.H63D	2.95 (11,214)	2.83 (4,199)	0.7472
White	≥55	p.C282Y	0.76 (9,911)	0.76 (1,192)	1.0000
White	≥55	p.H63D	0.83 (8,173)	0.55 (2,930)	0.1368
Asian	25–54	p.C282Y	3.12 (4,741)	12.50 (8)	0.2252
Asian	25–54	p.H63D	3.08 (4,345)	3.71 (404)	0.4562
Asian	≥55	p.C282Y	0.38 (2,864)	0.00 (2)	1.0000
Asian	≥55	p.H63D	0.38 (2,615)	0.40 (251)	1.0000
Pacific Islander	25–54	p.C282Y	4.49 (245)	11.11 (9)	0.3577
Pacific Islander	25–54	p.H63D	5.33 (225)	0.00 (29)	0.3706
Pacific Islander	≥55	p.C282Y	1.05 (190)	0.00 (5)	1.0000
Pacific Islander	≥55	p.H63D	1.09 (183)	0.00 (12)	1.0000
Native American	25–54	p.C282Y	7.36 (299)	0.00 (9)	1.0000
Native American	25–54	p.H63D	7.88 (241)	4.48 (67)	0.4303
Native American	≥55	p.C282Y	0.86 (116)	0.00 (11)	1.0000
Native American	≥55	p.H63D	0.97 (103)	0.00 (24)	1.0000
Other^b^	25–54	p.C282Y	4.39 (775)	2.00 (50)	0.7167
Other^b^	25–54	p.H63D	4.46 (672)	3.27 (153)	0.6582
Other^b^	≥55	p.C282Y	0.25 (399)	0.00 (25)	1.0000
Other^b^	≥55	p.H63D	0.28 (362)	0.00 (62)	1.0000

^a^Iron deficiency was defined as the combination of initial screening transferrin saturation <10% and initial screening serum ferritin <33.7 pmol/L (<15 μg/L). Values of p were obtained using Fisher’s exact test (two-tailed). NA = not available. *HFE* p.C282Y/p.H63D compound heterozygotes were counted once for each allele. Similar analyses that excluded p.C282Y/p.H63D compound heterozygotes revealed results consistent with those above (data not shown).

^b^Participants who reported two or more race/ethnicity groups or unknown race/ethnicity were classified as Other.

### Pregnancy in women with iron deficiency

Proportions of white and Native American women with ID who reported pregnancy were significantly greater than proportions of women of the corresponding race/ethnicity groups who reported no pregnancy (Table 3). The proportion of black women with ID who reported pregnancy was significantly lower than the proportion of black women who reported no pregnancy (Table 3). The proportion of Native American women with ID who reported pregnancy was significantly higher than the proportion of Native American women who reported no pregnancy, although the number of Native American women who reported pregnancy was relatively low (Table 3).

**Table 3:** Self-reported pregnancy in women ages 25–44 y with iron deficiency (ID)^a^.

Race/ethnicity	Pregnant with ID, % (n)	Not pregnant with ID, % (n)	Value of p
Hispanic	7.82 (563)	6.17 (5,430)	0.1447
Black	3.52 (369)	6.11 (10,109)	0.0438
White	4.41 (862)	2.82 (14,351)	0.0118
Asian	4.97 (181)	3.14 (4,262)	0.1907
Pacific Islander	6.25 (16)	4.44 (225)	0.5381
Native American	25.00 (20)	6.34 (268)	0.0118
Other^b^	3.57 (28)	4.17 (744)	1.0000

^a^ID was defined as the combination of initial screening transferrin saturation <10% and initial screening serum ferritin <33.7 pmol/L (<15 μg/L). Values of p were obtained using Fisher’s exact test (two-tailed). NA = not available.

^b^Participants who reported two or more race/ethnicity groups or unknown race/ethnicity were classified as Other.

### *HFE* p.C282Y and p.H63D alleles in iron-deficient women by pregnancy

The prevalence of p.C282Y and p.H63D alleles did not differ significantly in women with ID with or without pregnancy (Table 4). Comparisons on p.C282Y could not be made in Asian and Pacific Islander women because p.C282Y was not detected in these race/ethnicity subgroups (Table 4).

**Table 4:** *HFE* p.C282Y and p.H63D alleles in iron-deficient women by pregnancy^a^.

Race	Pregnancy	*HFE* allele	0 alleles, % (n subjects)	Any allele, % (n subjects)	Value of p
Hispanic	Yes	p.C282Y	7.79 (552)	9.09 (11)	0.5949
Hispanic	Yes	p.H63D	8.37 (466)	5.15 (97)	0.4045
Hispanic	No	p.C282Y	6.13 (5,257)	7.51 (173)	0.4220
Hispanic	No	p.H63D	6.00 (4,397)	6.87 (1,033)	0.3142
Black	Yes	p.C282Y	3.31 (362)	14.29 (7)	0.2237
Black	Yes	p.H63D	3.70 (351)	0.00 (18)	1.0000
Black	No	p.C282Y	6.08 (9,887)	7.66 (222)	0.3204
Black	No	p.H63D	6.10 (9,561)	6.39 (548)	0.7830
White	Yes	p.C282Y	4.90 (735)	1.57 (127)	0.1035
White	Yes	p.H63D	4.62 (628)	3.85 (234)	0.7119
White	No	p.C282Y	2.87 (12,456)	2.53 (1,895)	0.4567
White	No	p.H63D	2.88 (10,205)	2.68 (4,146)	0.5408
Asian	Yes	p.C282Y	4.97 (181)	NA	-
Asian	Yes	p.H63D	5.17 (174)	0.00 (7)	1.0000
Asian	No	p.C282Y	3.13 (4,254)	12.50 (8)	0.2257
Asian	No	p.H63D	3.11 (3,889)	3.49 (373)	0.6420
Pacific Islander	Yes	p.C282Y	6.25 (16)	NA	-
Pacific Islander	Yes	p.H63D	7.14 (14)	0.00 (2)	1.0000
Pacific Islander	No	p.C282Y	4.17 (216)	11.11 (9)	0.3408
Pacific Islander	No	p.H63D	5.00 (200)	0.00 (25)	0.6072
Native American	Yes	p.C282Y	26.32 (19)	0.00 (1)	1.0000
Native American	Yes	p.H63D	27.78 (18)	0.00 (2)	1.0000
Native American	No	p.C282Y	6.61 (257)	0.00 (11)	1.0000
Native American	No	p.H63D	6.93 (202)	4.55 (66)	0.7711
Other^b^	Yes	p.C282Y	3.70 (27)	0.00 (1)	1.0000
Other^b^	Yes	p.H63D	3.70 (27)	0.00 (1)	1.0000
Other^b^	No	p.C282Y	4.20 (690)	3.70 (54)	1.0000
Other^b^	No	p.H63D	4.36 (597)	3.40 (147)	0.8178

^a^Iron deficiency was defined as the combination of initial screening transferrin saturation <10% and initial screening serum ferritin <33.7 pmol/L (<15 μg/L). Values of p were obtained using Fisher’s exact test (two-tailed). NA = not available. *HFE* p.C282Y/p.H63D compound heterozygotes were counted once for each allele. Similar analyses that excluded p.C282Y/p.H63D compound heterozygotes revealed results consistent with those above (data not shown).

^b^Participants who reported two or more race/ethnicity groups or unknown race/ethnicity were classified as Other.

### Effect of field center

We detected no evidence of an effect of *HFE* genotypes either overall or by Field Center in any race/ethnicity group. In analyses limited to women who reported pregnancy, in the subsets for which sample sizes allowed a test of Field Center effect, we detected no significant differences.

## Discussion

This HEIRS substudy of 62,685 women represents the largest cohort of women studied for ID in the US and Canada. The prevalence of ID in women of all race/ethnicity groups was greatest in women ages 25–54 y, consistent with observations on women ages 20–49 y in the National Health and Nutrition Examination Survey (NHANES) 1999–2000 [8] and the lower prevalence of sufficient SF (≥38 μg/L) in Canadian women ages 12–49 y than older women [24]. Among the present women ages 25–54 y, the prevalence of ID was significantly greater in Hispanic and black women than white and Asian women. In NHANES 1999–2000 and NHANES 2003–2010, the prevalence of ID was greater among non-Hispanic black and Mexican-American women than among non-Hispanic white women [8, 25]. Among US women ages 22–44 y identified as Mexican Americans, Puerto Ricans, or Cubans, the prevalence of low iron status was greatest in Mexican Americans [26]. Epidemiologic and clinical studies demonstrate that risk of ID is greatest in women of reproductive age. The predominant causes of ID in pre-menopausal women are iron losses of menstruation, pregnancy, and lactation, although the risk of ID is also increased in women who have menorrhagia, donate blood, or receive high-dose aspirin therapy [2]. Women with these conditions may benefit from iron supplementation [2].

Race/ethnicity differences in onset and duration of menses could explain some of the present observations. Menarche occurs at a significantly earlier age in non-Hispanic black girls than non-Hispanic white [27, 28] and Mexican-American [27] girls. US-born Asian Americans had mean age at menarche of 12.1 y, similar to that in whites, and 1.4 y earlier than Asian women who migrated to the US [29]. Mean duration of bleeding was 5.1 d for African-American and 5.6 d for European-American girls ages 12–14 y [30]. The mean cycle length for European-American and African-American girls ages 12–14 y was 29.3 d and 28.8 d, respectively [31]. In a community-based, longitudinal Study of Women’s Health Across the Nation, US Hispanic women ages 42–52 y had more long cycles (≥33 d) and fewer short cycles (≤22 d) than US Caucasian, African-American, Chinese, and Japanese women ages 42–52 y [32].

There are differences in numbers of children per mother classified by race/ethnicity. Among US women ages 40–44 y, Hispanic, black, white, and Asian mothers have had an average of 2.6, 2.5, 2.3, and 2.2 children, respectively [33]. Fifty percent of Hispanic mothers ages 40–44 y have had three or more children. This proportion is higher than that of blacks (40%), whites (33%), and Asians (27%) [33]. These observations are consistent with the greater prevalence of ID in the present Hispanic and black women ages 25–54 y than white and Asian women ages 25–54 y.

There are race/ethnicity differences in breastfeeding. In a population-based study, the proportion of Asian-American women who breastfed was slightly higher than that previously reported in US white women [29]. In the 2004 National Immunization Survey, 50% of non-Hispanic black children and 72% of US non-Hispanic white children were ever breastfed. Among those ever breastfed, 43% of non-Hispanic black children and 54% of non-Hispanic white children continued breastfeeding until at least age 6 months [34]. The duration of breastfeeding was similar in US-born Asians [29]. Among major US race/ethnicity groups, Asian and Hispanic women achieve the highest rates of breastfeeding initiation and continuation at 6 months and 12 months. African-American women have the lowest rates [35]. The iron content of human breast milk is 0.5–1.0 mg/L [36, 37]. The quantity of iron lost by lactation is 0.5–1.0 mg daily [2]. Increasing duration of breast feeding of U.S. children is associated with increasing risk of decreased iron stores or ID [38, 39], although the iron content of human breast milk is probably not affected by maternal iron stores [40]. Estimating the effect of lactation on ID risk must also consider the average numbers of children born to mothers of respective race/ethnicity groups.

In this study, the prevalence of ID in women ages ≥55 y was lower than that of younger women and did not differ significantly across race/ethnicity groups. The prevalence of ID in Framingham residents ages 67–96 y was also low [41]. In NHANES 1988–1994, SF levels increased in women after menopause. The increase was more rapid and maximum SF levels were greater for black women than white and Hispanic women of similar ages [7]. In the HEIRS Study, TS and SF began to increase at a younger age in black than white women, and the rate of increase of both TS and SF was greater in black than white women [23]. The most common cause of ID in post-menopausal women is gastrointestinal blood loss [2].

Dietary intake did not account for differences in low iron stores among Mexican-American and non-Hispanic white women who participated in NHANES 1988–1994 [42]. In NHANES 2003–2006 participants, non-Hispanic blacks had lower dietary iron intake than non-Hispanic whites and Mexican Americans, but supplemental iron intake was unrelated to race/ethnicity [43].

*Helicobacter pylori* gastritis increases the risk of ID with or without peptic ulcer and the risk increases with increasing age [44, 45]. Analyses of NHANES 1988–1991 data revealed that antibody positivity for *H*. *pylori* is associated with a 40% increase in the prevalence of ID, after adjustment for other pertinent factors [46], although the specificity of antibody testing for active infection is <80% [47]. Among healthy asymptomatic residents of the Houston metropolitan area ages 15–80 y, the prevalence of *H*. *pylori* infection was twice as great in blacks as whites by ^13^C-urea breath testing [48]. The sensitivity and specificity for active infection typically exceed 95% [47]. The age-adjusted prevalence of antibody positivity for *H*. *pylori* was 62% in Mexican Americans, 53% in non-Hispanic blacks, and 26% non-Hispanic whites in NHANES 1988–1991 [44]. US Hispanics and Native Americans/Alaskan Natives have higher rates of *H*. *pylori* antibody positivity than whites [49, 50]. In HEIRS Study participants without ID in another report, the prevalence of *H*. *pylori* antibody positivity was significantly greater in Asian/Pacific Islander participants than control subjects [51]. The prevalence of *H*. *pylori* antibody positivity did not differ significantly between white, black, and Hispanic participants with or without ID, respectively [51].

The prevalence of celiac disease in the US is 0.71% (95% confidence interval: 0.58–0.86%) and in US non-Hispanic whites is 1.01% (95% confidence interval: 0.78–1.31%) [52]. Some persons with celiac disease also have ID [53]. In a HEIRS substudy, the prevalence of celiac disease was significantly greater in whites than non-whites, and the odds of celiac disease in men ages ≥25 y and women ages ≥50 y participants with SF ≤12 μg/L was 28-fold higher than that of controls [54]. Thus, celiac disease is associated with an increased prevalence of ID in women, especially white women.

It was hypothesized that hemochromatosis heterozygosity, later defined as *HFE* p.C282Y heterozygosity, increases iron absorption and thereby decreases risk of iron-deficiency anemia, especially in women [17, 18], or that p.C282Y became common in Neolithic Europeans as an adaptation to low dietary iron content [55]. In the present analyses, the prevalence of ID in female participants in the HEIRS Study with and without p.C282Y did not differ significantly, regardless of race/ethnicity or age subgroup. In female Welsh blood donors, mean SF levels and proportions of women with SF <15 μg/L did not differ significantly between those with p.C282Y heterozygosity and *HFE* wild-type genotypes [56]. In Australian women, mean SF levels and prevalence of SF <12 μg/L did not differ significantly between those with p.C282Y heterozygosity and *HFE* wild-type genotypes [57]. In a California health appraisal clinic cohort, the prevalence of ID without anemia was lower among women with p.C282Y heterozygosity, but the prevalence of iron-deficiency anemia did not differ significantly between men and women with or without p.C282Y [58]. In female Danish blood donors, logistic regression on SF <15 μg/L did not reveal a significant association with p.C282Y, after adjustment for other variables [59]. In a logistic regression on SF <15 μg/L in non-pregnant, non-lactating Hispanic, black, white, and Asian women in the HEIRS Study, there was no significant association with p.C282Y, after adjustment for other variables [60]. Taken together, these observations indicate that p.C282Y heterozygosity has no significant association with ID in women.

HFE protein is one of many upstream regulators of hepcidin transcription and consequent iron absorption [13]. Hepcidin levels and hepcidin/ferritin ratios are similar in adults with *HFE* p.C282Y heterozygosity and wild-type genotypes [61]. p.C282Y heterozygotes do not absorb dietary heme or non-heme iron more efficiently than control subjects [62]. Non-heme iron absorption in men with p.C282Y heterozygosity, p.C282Y/p.H63D compound heterozygosity, or *HFE* wild-type genotypes did not differ significantly [63]. In healthy pregnant women unselected for *HFE* genotypes, hepcidin levels are suppressed in the second and third trimesters due to incompletely defined mechanisms and contribute to increased iron absorption [64, 65]. In pregnant women with obesity or other inflammatory disorders, hepcidin levels may be inappropriately high and thus decrease iron absorption [64, 65].

In the present study, the prevalence of p.C282Y and p.H63D alleles did not differ significantly in women with ID with or without self-reported pregnancy. Respective comparisons of the prevalence of p.C282Y in Asian and Pacific Islander women with ID with or without self-reported pregnancy could not be made because p.C282Y was not observed in these race/ethnicity subgroups. In another study, logistic regression on SF <15 μg/L in pregnant or lactating women revealed a significant inverse association with p.C282Y, after adjustment for other factors [60]. Thus, it is plausible but unproven that a subtle effect of p.C282Y heterozygosity or associated non-*HFE* genetic or acquired modifiers influence iron absorption during pregnancy or lactation.

In this study, the prevalence of ID in women with or without *HFE* p.H63D did not differ significantly, regardless of race/ethnicity or age subgroup. In female blood donors in Wales, mean SF levels did not differ significantly between p.H63D heterozygotes, p.H63D homozygotes, and donors with *HFE* wild-type genotypes [56]. In a California health appraisal clinic cohort, the overall prevalence of ID without anemia was significantly lower in women with p.H63D heterozygosity and in women ages <50 y with either p.H63D heterozygosity or homozygosity [58]. In female Danish blood donors, logistic regression on SF <15 μg/L revealed a significant negative association with p.H63D homozygosity but not heterozygosity, after adjustment for other variables [59].

The prevalence of ID in the present Asian women ages 25–54 y was low. The T allele at tag rs9366637 (*HFE* intron 1, C>T) captured 96% of a haplotype that is common in Asians (54%) but uncommon in Northwestern Europeans (6%) and East Africans (4%) [66]. Iron absorption in Asian-American women homozygous for rs9366637 (T/T) was significantly greater than that of Asian-American women without rs9366637 (C/C) or age-matched Caucasian-American women [66]. Thus, positivity for rs9366637 could partly explain the low prevalence of ID in the present Asian women ages 25–54 y.

Advantages of this study include the availability of observations for race/ethnicity, age, TS, SF, *HFE* p.C282Y and p.H63D, and self-reported pregnancy at a single screening encounter in a large cohort of women who reside in the US and Canada with complete data, although overall numbers of Pacific Islander, Native American, and Other participants were small.

A major goal of the present study was to determine the association of ID (defined as combined TS <10% and SF <15 μg/L) with common *HFE* alleles. Reduction or absence of storage iron in the bone marrow and hepatocytes is associated with a continuum of laboratory and clinical abnormalities, only the most severe of which are typically classified as ID (with or without anemia) [2]. We sought to minimize the numbers of women (pregnant and non-pregnant) classified as having ID who were "false-positives." Accordingly, we used a stringent definition of ID consistent with general population survey studies [1, 2, 20, 21]. In other studies that evaluated the associations of ID with common *HFE* alleles, ID was defined as SF <12 μg/L [57] or SF <15 μg/L [56, 59, 60]. We acknowledge that the present definition of ID could result in an underestimate of the proportion of women with iron depletion or ID, although ancillary laboratory measures including estimates of stainable iron in bone marrow, levels of hemoglobin, erythrocytes, serum transferrin receptor, and erythrocyte protoporphyrin typically used to diagnose or classify pre-latent or latent ID [2] were not available as part of HEIRS Study initial screening tests.

A limitation of using HEIRS Study initial screening data to evaluate women for ID is the inability to determine causation of ID in individual women. Socio-economic assessments, clinical evaluations, management of ID and its underlying causes, and longitudinal follow-up of participants were beyond the scope of the HEIRS Study. We did not evaluate age of menarche, quantify reproductive (menses, pregnancy, lactation) and non-reproductive iron losses, or perform analyses to detect uncommon *HFE* or non-*HFE* iron-related mutations.

## Conclusions

ID prevalence was greater in Hispanic and black than white and Asian women ages 25–54 y. p.C282Y and p.H63D prevalence did not differ significantly in women with or without ID, regardless of race/ethnicity, age subgroup, or pregnancy. The present results may inform recommendations by public health officials and epidemiologists concerning screening women with increased risk of ID, regardless of race/ethnicity.
